# Ceftibuten-polymyxin B combination alters resistance and cell wall gene expression in multidrug-resistant *Klebsiella pneumoniae*

**DOI:** 10.1371/journal.pone.0349583

**Published:** 2026-06-05

**Authors:** Mariana Carvalho Sturaro, Ruana Carolina Cabral da Silva, Simone Simionatto

**Affiliations:** Federal University of Grande Dourados (UFGD), Health Sciences Research Laboratory, Dourados, Mato Grosso do Sul, Brazil; University of Pennsylvania Perelman School of Medicine, UNITED STATES OF AMERICA

## Abstract

**Background:**

Multidrug-resistant *Klebsiella pneumoniae*, including carbapenem- and polymyxin-resistant strains, poses a major public health threat by severely limiting therapeutic options. Although ceftibuten (CTB) and polymyxin B (PMB) show evidence of synergistic activity, their mechanisms of action remain unclear. In this context, this study evaluated the combined effect of CTB and PMB on the expression of genes involved in cell wall synthesis and antimicrobial resistance in *K. pneumoniae*.

**Methods:**

Protein sequences of selected resistance- and cell wall-related genes were retrieved from UniProt and analyzed *in silico* for conserved domains, motifs, localization (PSORTb), and pathways (KEGG). The effects of CTB/PMB, alone and in combination, were evaluated on the expression of genes involved in cell wall synthesis regulation (*ftsL, dacA,* and *dacC*) and antimicrobial resistance mechanisms (*pagP, pagL, ampC)*. The 16s gene was used as endogenous control. Bacterial cultures (1.5 × 10^6^ CFU/mL) were incubated with CTB/PMB, PMB and CTB at 0.5× and 1 × minimum inhibitory concentrations (MIC) for 2 and 4 h, followed by mRNA extraction and gene expression analysis through qRT-PCR.

**Results:**

*In silico* analyses revealed that *pagP* and *pagL* contain conserved domains associated with lipid A modification, while *ampC* and *ftsL* showed class C β-lactamase domains. *DacA* and *dacC* were linked to the PRK13482 domain of the cl44310 superfamily. Predicted localizations placed *pagP/pagL* in the outer membrane, *ampC* in the periplasm, and *ftsL/dacA/dacC* in the cytoplasmic membrane. KEGG annotation indicated that *ftsI*, *dacC*, *ampC*, and *rtxB* participate in essential pathways, including peptidoglycan biosynthesis and β-lactam resistance, highlighting their physiological and clinical relevance in *K. pneumoniae*. After 2 h of exposure, no significant changes were observed in the expression of the selected genes. However, after 4 h, *dacA, ftsL,* and *pagP* expression levels significantly increased in all groups treated with CTB/PMB. Additionally, *dacC* and *pagL* expression were upregulated in the group treated with the combination at 1 × MIC.

**Conclusions:**

Thus, combined CTB/PMB exposure was associated with modulation of gene expression involved in cell wall synthesis and antimicrobial resistance, suggesting transcriptional adaptive responses under antimicrobial pressure. These findings provide preliminary molecular insights into the response of multidrug-resistant *K. pneumoniae* to CTB/PMB exposure.

## 1. Introduction

Antimicrobial resistance (AMR) in Gram-negative bacilli represents a major clinical concern, particularly in carbapenem- and polymyxin-resistant *Klebsiella pneumoniae* (CPR-*Kp*), which has been recognized by the World Health Organization as a critical priority pathogen for the development of new antibiotics [1,2]. The convergence of plasmid-borne resistance determinants (e.g., *bla*_*KPC*_*, bla*_*NDM*_*, bla*_*OXA-48-like*_) with chromosomal alterations in porins (ompK35/ompK36) and envelope regulatory systems (e.g., PhoPQ, PmrAB) underpins multidrug-resistant phenotypes, drastically reducing therapeutic options [3].

In this context, pharmacological combinations have been explored to assess potential antimicrobial synergy and to investigate genetic responses that might partially reverse extreme resistance profiles [4]. Among β-lactams, ceftibuten (CTB) occupies a peculiar role: although its stability is limited in the presence of extended-spectrum β-lactamases (ESBLs) and AmpC enzymes, its action on cell wall targets may remodel the bacterial envelope and, theoretically, modulate permeability to amphipathic agents such as polymyxin B (PMB) [5].

Resistance to polymyxins in *K. pneumoniae* is mainly associated with *mgrB* inactivation by insertion sequences or to mutations in the *phoPQ* and *pmrAB* systems, which induce lipopolysaccharides (LPS) modifications through the arnBCADTEF and eptA operons, ultimately reducing drug affinity [6]. However, how multidrug-resistant *K. pneumoniae* modulates gene expression when simultaneously exposed CTB and PMB remains poorly understood [7]. Understanding these adaptive responses is essential to elucidate key mechanisms underlying cell wall synthesis, envelope remodeling, and antimicrobial resistance regulation. In this study, we aimed to evaluate the effect of CTB/PMB combination on the expression of genes involved in the regulation of cell wall synthesis and antimicrobial resistance in CPR-Kp.

## 2. Methodology

### 2.1. *In silico* analyses

To select the targets, a search was conducted for curated protein sequences of *pagP*, *pagL*, *ampC*, *ftsL*, *dacA*, and *dacC* in the Universal Protein (UniProt) database (https://www.uniprot.org/). These genes were chosen based on evidence from the literature highlighting their central roles in antimicrobial resistance and cell wall remodeling in *K. pneumoniae* and other Gram-negative pathogens. After retrieving the FASTA sequences, conserved domains and motifs were identified using Batch CD-Search (https://www.ncbi.nlm.nih.gov/Structure/bwrpsb/bwrpsb.cgi) and MEME Suite 5.5.8 (https://meme-suite.org/meme/tools/meme), respectively. Subsequently, subcellular localization was verified using PSORTb v3.0 (https://psort.org/psortb/), and the involvement of each protein in specific cellular processes was mapped through the Kyoto Encyclopedia of Genes and Genomes (KEGG) (https://www.genome.jp/kegg/). This workflow ensured that the selected targets were not only supported by biological relevance described in the literature but also validated by computational analysis for their structural and functional importance.

### 2.2. Microrganisms and cultivation conditions Chemicals

One CPR-Kp strain was used in the experiment [8]. Briefly, the bacterial culture was prepared at a concentration of 1.5 × 10^6^ CFU/mL and subsequently divided into five experimental groups: Group 1- CTB at 0.5 × minimum inhibitory concentration (MIC); Group 2- PMB at 0.5 × MIC; Group 3- CTB/PMB at 0.5 × MIC; Group 4- CTB/PMB at 1 × MIC; and Group 5- untreated bacteria. The MIC values were 4 mg/L for PMB and 2 mg/L for CTB, and the combination exhibited a fractional inhibitory concentration index (FICI) of 0.15, indicating a synergistic interaction between the drugs (Sturaro et al., 2025). The experimental design considered both the individual and combined effects of the compounds under different exposure conditions (S1 Table).

Treatment was initiated at time 0 h, and samples were collected at two predefined time points (2 h and 4 h), selected based on prior time–kill experiments demonstrating that the drug combination achieved bacterial killing within 4 h. The selected time points (2 h and 4 h) were chosen to capture early transcriptional responses to antimicrobial exposure. Experiments were performed with three biological replicates per group. At the end of each exposure period, bacterial cultures were centrifuged at 500 × g for 5 min at 4 °C, and the supernatants were discarded. The resulting pellets were immediately frozen at −80 °C. Additionally, an aliquot of the *K. pneumoniae* culture was sent in parallel for RT-qPCR reaction standardization, enabling the analysis of gene expression related to cell wall synthesis and envelope integrity.

### 2.3. *Primer* design and validation

Specific primers targeting selected genes involved in cell wall synthesis and antimicrobial resistance were designed and optimized for this study. Primer sequences were generated using Primer3 software. Primers were synthesized and standardized with annealing temperatures optimized via gradient pPCR. Efficiency was assessed using standard curves from serial cDNA dilutions, and specificity was confirmed by melting curve analysis. Primer sequences and characteristics are provided in S2 Table.

### 2.4. qRT-PCR

In order to identify the possible mechanisms of action of CTB/PMB and their role in modulating cell wall synthesis, qRT-PCR was employed to evaluate gene expression. For this purpose, total RNA from CPR-Kp cultures treated with the combination was obtained by using RNA affinity columns (PureLink™ RNA Mini Kit, Thermo Fisher Scientific, Waltham, MA), being subsequently subjected to DNA digestion (PureLink™ DNase Kit, Thermo Fisher Scientific, Waltham, MA) to avoid any interference. Subsequently, cDNA synthesis was carried out using the High-Capacity™ cDNA Reverse Transcription Kit (Thermo Fisher Scientific, Waltham, MA). qRT-PCR was performed using PowerUp™ SYBR™ Green Master Mix (TaqMan™ system, Thermo Fisher Scientific).

The analysis was performed on a StepOne Plus™ Real-Time PCR System (Thermo Fisher Scientific, Waltham, MA), and relative gene expression was calculated using the 2^(-ΔΔCt) method, normalized to the 16S endogenous control gene, where:


$$\begin{array}{cc} \Delta \Delta Ct= & \;\left[(Ct\;target\;gene\;-\;Ct\;endogenous\;control)\;sample\;analyzed\right]\;\\ & -\;\left[(Ct\;target\;gene\;-\;Ct\;endogenous\;control)\;normalizer\;sample\right] \end{array}$$

mRNA expression values were expressed relative to the control group as fold change.

The 16S rRNA gene was used as the endogenous control for normalization. Its stability was not formally validated under all experimental conditions used in this study.

### 2.5. Statistical analysis

GraphPad Prism software (GraphPad Software Inc., San Diego, CA, USA) was used for statistical analyses. For parametric data with only one variable, the Student’s t-test was applied. One-way analysis of variance (ANOVA), followed by Dunnett’s multiple comparisons test, was used to evaluate differences between each treatment group and the control condition. A p-value < 0.05 was considered statistically significant.

## 3. Results

**Fig 1:**
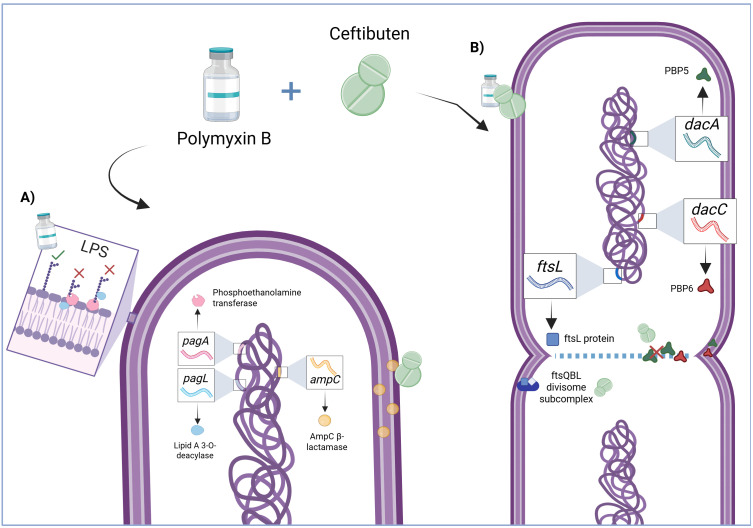
Schematic representation of the predicted subcellular localization of proteins associated with antimicrobial resistance (A) and cell wall synthesis (B) in CPR-Kp. The localization was inferred using bioinformatic analysis (PSORTb v3.0). This figure is provided for illustrative purposes only and does not represent experimental validation.

The analysis of conserved domains and motifs revealed that *pagP* and *pagL* possess complete and highly conserved domains directly associated with the lipid A palmitoyltransferase PagP. *AmpC* and *ftsL* presented domains directly related to class C β-lactamases, with domain coverage across the entire protein. For *dacA* and *dacC*, the domain PRK13482 was identified; although it lacks a directly interpretable short name, it represents a specific protein within the cl44310 superfamily. It was observed that *pagP* and *pagL* proteins (PagP palmitoyl transferase and Lipid-A 3-O-deacylase, respectively) are located in the outer membrane, while AmpC is localized in the periplasmic space, and FtsL, DacA, and DacC are anchored in the cytoplasmic membrane (Fig 1).

Functional annotation analyses using KEGG revealed that the genes are associated with important metabolic pathways related to physiology and AMR in *K. pneumoniae*, with *ftsI*, *dacC*, *ampC*, and *rtxB* directly involved in general metabolic pathways (map01100); peptidoglycan biosynthesis (map00550); and β-lactam resistance (map01501). These analyses provide functional context for the selected genes but do not directly explain the transcriptional responses observed.

**Fig 2:**
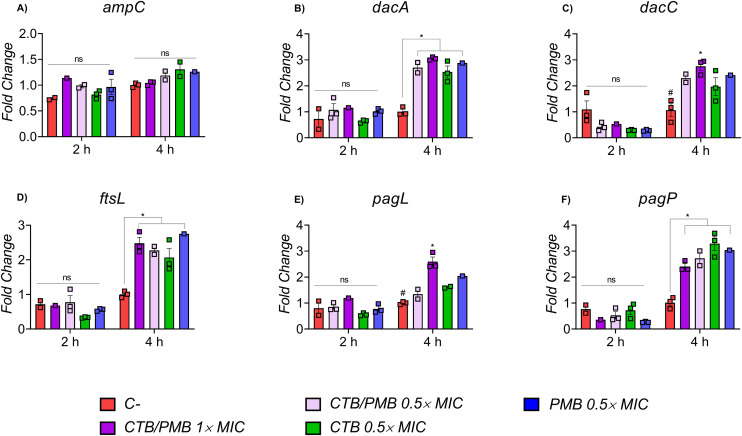
Experimental qRT-PCR analysis of gene expression in CPR-Kp after 2 and 4 h of exposure to CTB/PMB. The experimental groups included: CPR-Kp + vehicle (C−); CPR-Kp + CTB/PMB 0.5 × MIC; CPR-Kp + CTB/PMB 1 × MIC; CPR-Kp + CTB 0.5 × MIC; and CPR-Kp + PMB 0.5 × MIC. mRNA expression levels were normalized to the endogenous 16S gene, and relative expression was calculated using the 2^ − ΔΔCt method, expressed as fold change relative to the control group. Gene expression profiles are shown for (A) ampC, (B) dacA, (C) dacC, (D) ftsL, (E) pagL, and (F) pagP. Data represent three biological replicates. The Ct values for the 16S reference gene were 23.9 at 2 h and 22.2 at 4 **h.** MIC: minimum inhibitory concentration.

In contrast, *amp*C expression remained stable under all conditions, suggesting that β-lactamase-mediated resistance in this strain is not transcriptionally modulated by CTB/PMB exposure. Expression analysis showed that *ampC* remained stable across all groups after 2 and 4 h of treatment (Fig 2A). Similarly, the *dacA* gene, which encodes D-alanine carboxypeptidase (PBP5), gene associated with peptidoglycan synthesis, exhibited no significant changes in expression in response to the Test Items compared with the vehicle group in 2 h, however had increased expression in all treatment groups within 4 h (Fig 2B). A similar pattern was observed for the *ftsL* gene, a crucial component of the bacterial divisome, which has been previously associated with septum formation during cytokinesis during cytokinesis and consequently affect cell viability (Fig 2D). The *dacC* gene, encoding PBP6, another enzyme involved in peptidoglycan biosynthesis, displayed an increased expression in CTB/PMB 1 × MIC group after 4 h (Fig 2C).

Regarding genes involved in outer membrane remodeling, *pagL*, which encodes an enzyme that modifies LPS and has been linked to polymyxin resistance, showed significant variation in expression between CTB/PMB 1 × MIC group and the control after 4 h (Fig 2E). Conversely, *pagP*, which also modifies LPS and has been implicated in adaptation to polymyxin exposure, demonstrated a consistent trend toward increased transcription in all groups exposed to the Test Items after 4 h (Fig 2F).

## 4. Discussion

The study evaluated the effects of CTB and PMB under sub-inhibitory and inhibitory concentrations to assess modulation of key pathways, including genes associated with expression of porins, efflux systems, and envelope remodeling. Results obtained in this study showed a biphasic and time-dependent response to the antimicrobial pressure exerted by CTB and PMB. Within the first 2 h, transcriptional changes were minimal, suggesting predominance of basal homeostasis and rapid post-transcriptional adjustments predominate. However, after 4 h, clear modulation patterns emerged, characterized by the induction of *dacA* and *ftsL* in all treated groups, increased expression of *dacC* and *pagL* in the CTB/PMB 1 × MIC group, and *pagP* was consistently upregulated. This finding showed that the transcriptional adaptation of resistant *K. pneumoniae* requires sustained exposure, corroborating previous studies describing the temporal plasticity of bacterial responses to antimicrobial combinations [9,10].

The *in silico* analyses provided the structural and functional foundation to interpret these findings. The predicted subcellular localization of *pagP* and *pagL* proteins in the outer membrane reinforces their role in lipid A modification, an essential component of LPS. In this study, the increased expression of *pag*P and *pag*L after 4 h suggests activation of transcriptional responses associated with outer membrane remodeling under CTB/PMB exposure. This pattern is consistent with previously described roles of these genes in lipid A modification, although no direct functional assessment was performed, similar mechanisms have been described in *Pseudomonas aeruginosa* [11] and *Escherichia coli* [12]. Although not directly investigated in the present study, the late induction of these genes may indicate activation of LPS remodeling pathways, potentially driven by regulatory systems such as PhoPQ/PmrAB, which has been described as a key pathway in the adaptation of *K. pneumoniae* to polymyxin pressure [6]. These analyses provide functional context for the selected genes but do not directly explain the transcriptional responses observed.

Complementarily, genes associated with peptidoglycan synthesis and remodeling also corroborated the bioinformatic predictions. Specifically, *dacA* and *dacC*, linked to the cl44310 superfamily and to cell wall biosynthesis pathways in KEGG, exhibited increased expression after 4 hours, with *dacC* showing particular induction in the CTB/PMB 1 × MIC group. Likewise, the upregulation of *dac*A, *dac*C, and *fts*L after 4 h suggests transcriptional modulation of genes involved in cell wall synthesis and division under antimicrobial pressure. This response may reflect an adaptive adjustment to envelope stress [13], although the functional consequences of these changes were not assessed in the present study. Notably, the increased expression of *fts*L further supports the involvement of cell division-associated pathways during CTB/PMB exposure. However, the absence of analysis of central divisome regulators, such as *ftsZ*, precludes more definitive conclusions regarding divisome dynamics [14]. Considering that CTB exerts its antibacterial effect by targeting penicillin-binding proteins involved in septal peptidoglycan cross-linking, the observed transcriptional response may be associated with cellular processes directly affected by CTB in *K. pneumoniae* [15]. These findings warrant further investigation to clarify whether this late transcriptional induction represents a compensatory stress response, altered divisome regulation, or a broader envelope adaptation mechanism under combined CTB/PMB exposure.

In the case of *ampC*, the data demonstrated expression stability at both 2 h and 4 h, regardless of the treatment condition. This result indicates that resistance mediated by class C β-lactamase is already established in the isolate and is not transcriptionally modulated by CTB/PMB, in line with reports of constitutive AmpC-mediated resistance [16]. This stability contrasts with the plasticity observed in cell wall and LPS-related genes, reinforcing that the main adaptive response under combination pressure involves structural mechanisms rather than β-lactamase amplification.

Functional annotation results from KEGG further highlighted the overlap among cell wall biosynthesis, β-lactam resistance, and LPS remodeling pathways. This convergence demonstrates the interdependence of structural maintenance, resistance mechanisms, and virulence factors, an aspect previously emphasized in integrative genomic and transcriptomic analyses of *K. pneumoniae* [17]. Moreover, recent studies have pointed out that the complexity of resistance in multidrug-resistant Gram-negative pathogens requires the exploration of new β-lactam/inhibitor combinations [18] and the development of innovative approaches that consider the adaptive plasticity of the bacterial envelope [19].

The KEGG-based functional annotation supports the biological relevance of the selected genes; however, these data provide contextual information and do not directly explain the transcriptional responses observed in this study.

This study has several limitations that should be considered when interpreting the findings. First, the analysis was conducted using a single *K. pneumoniae* strain, which restricts the extent to which these results can be generalized to other resistant isolates or strains belonging to different sequence types. Second, only two antibiotic-exposure time points were evaluated, limiting the ability to capture broader temporal dynamics and potentially transient transcriptional responses.

In addition, 16S rRNA was used as the sole endogenous control for qRT-PCR normalization, although its stability was not formally validated under all antibiotic-stress conditions examined here. This aspect should be taken into account when interpreting the normalized transcriptional data. Moreover, changes in transcript levels do not necessarily translate into corresponding alterations in protein abundance or activity, since bacterial adaptive responses may also be shaped by post-transcriptional and post-translational regulatory mechanisms. Finally, relevant physiological parameters, including bacterial viability, membrane integrity, morphological alterations, and lipid A remodeling, were not investigated and warrant further study to better contextualize the transcriptional patterns observed.

In conclusion, combined exposure to CTB and PMB was associated with transcriptional changes in multidrug-resistant *K. pneumoniae*, particularly in genes involved in cell wall synthesis and lipopolysaccharide modification, while *ampC* remained stable. These findings suggest activation of adaptive responses under antimicrobial pressure and provide preliminary molecular data for future studies on the mechanisms of this combination.

## Supporting information

S1 Table Representation of experimental groups, collection times and experimental number. (DOCX)

S2 Table Brief description of the genes that were analyzed in this study and nucleotide sequence. (DOCX)
