# Supplementary material for: Ceftibuten-polymyxin B combination alters resistance and cell wall gene expression in multidrug-resistant Klebsiella pneumoniae
Source: PLoS One. 2026 Jun 5;21(6):e0349583. doi: 10.1371/journal.pone.0349583 (PMC13240885; doi:10.1371/journal.pone.0349583)
Supplement: S1 Table — (DOCX) [file pone.0349583.s001.docx]

**Table S1.** Representation of experimental groups, collection times and experimental number.

| **Experimental groups** | | | | | |
| --- | --- | --- | --- | --- | --- |
| Experimental groups | Group 1 | Group 2 | Group 3 | Group 4 | Group 5 |
| Test Item | CTB  (0,5 MIC) | PMB  (0,5 MIC) | CTB/PMB (0,5 MIC) | CTB/PMB (1 MIC) | Untreated bacteria |
| Sample collection times | 2 and 4 hours | 2 and 4 hours | 2 and 4 hours | 2 and 4 hours | 2 and 4 hours |
| Trial No. | N = 3 per group for each time evaluated | | | | |

(MIC) Minimum inhibitory concentration.
