# Supplementary material for: Ceftibuten-polymyxin B combination alters resistance and cell wall gene expression in multidrug-resistant Klebsiella pneumoniae
Source: PLoS One. 2026 Jun 5;21(6):e0349583. doi: 10.1371/journal.pone.0349583 (PMC13240885; doi:10.1371/journal.pone.0349583)
Supplement: S2 Table — (DOCX) [file pone.0349583.s002.docx]

**Table S2.** Brief description of the genes that were analyzed in this study and nucleotide sequence.

| **Gene** | **Main functions** | **Primer Forward**  **5´-3´ Sequence** | **Reverse Primer**  **5´-3´ Sequence** |
| --- | --- | --- | --- |
| *ampC* | Beta-lactamase induced in response to cell wall dysfunction | GCGCATCCGGAGGGTATTTA | AGCGACCCTTTAAGCCCTTC |
| *dacA* (PBP5) | Essential for peptidoglycan synthesis | TCAACCTGCAGTCCGGTAAC | AGGGCGTTGACGTAGTTGTT |
| *dacC* (PBP6) | Essential for peptidoglycan synthesis | AAATTGCTGACCTGGGGCTT | TGCTATCGCCAAACCAGACG |
| *ftsl*  (PBP3) | Cell elongation and division | TGAACAGCTGGTTCTGGAGC | CGCTCAACCCGACTGTGAT |
| *pagL* | Resistance to antimicrobial peptides | GGCGGCAGTACTTATACCCC | ACGCTGGTGATGGTTGTCAT |
| *pagP* | Resistance to antimicrobial peptides | CAGAATTTCCACCTCGGCCT | GACGCCAATGGCAAGATCAC |
| *16S* | Constitutive gene - ribosomal | GCCCCCTCTACAGCAGAAAG | TTGCTGATGAGCCACTCCAG |
